# Synchronization and variability imbalance underlie cognitive impairment in primary-progressive multiple sclerosis

**DOI:** 10.1038/srep46411

**Published:** 2017-04-21

**Authors:** Maria Petracca, Catarina Saiote, Heidi A. Bender, Franchesca Arias, Colleen Farrell, Paola Magioncalda, Matteo Martino, Aaron Miller, Georg Northoff, Fred Lublin, Matilde Inglese

**Affiliations:** 1 Department of Neurology, Icahn School of Medicine at Mount Sinai, New York, 10029, NY, USA.; 2 Department of Neuroscience, Reproductive Sciences and Odontostomatology, University of Naples “Federico II”, Naples, 80131, Italy; 3 Department of Neuroscience, Rehabilitation, Ophthalmology, Genetics, and Mother-Child health, University of Genoa, Genoa, 16132, Italy; 4 Institute of Mental Health Research, University of Ottawa, Ottawa, K1Z 7K4, Canada; 5 Department of Radiology, Icahn School of Medicine at Mount Sinai, New York, 10029, NY, USA.; 6 Department of Neuroscience, Icahn School of Medicine at Mount Sinai, 10029, NY, USA

## Abstract

We aimed to investigate functional connectivity and variability across multiple frequency bands in brain networks underlying cognitive deficits in primary-progressive multiple sclerosis (PP-MS) and to explore how they are affected by the presence of cortical lesions (CLs). We analyzed functional connectivity and variability (measured as the standard deviation of BOLD signal amplitude) in resting state networks (RSNs) associated with cognitive deficits in different frequency bands in 25 PP-MS patients (12 M, mean age 50.9 ± 10.5 years) and 20 healthy subjects (9 M, mean age 51.0 ± 9.8 years). We confirmed the presence of a widespread cognitive deterioration in PP-MS patients, with main involvement of visuo-spatial and executive domains. Cognitively impaired patients showed increased variability, reduced synchronicity between networks involved in the control of cognitive macro-domains and hyper-synchronicity limited to the connections between networks functionally more segregated. CL volume was higher in patients with cognitive impairment and was correlated with functional connectivity and variability. We demonstrate, for the first time, that a functional reorganization characterized by hypo-synchronicity of functionally-related/hyper-synchronicity of functionally-segregated large scale networks and an abnormal pattern of neural activity underlie cognitive dysfunction in PP-MS, and that CLs possibly play a role in variability and functional connectivity abnormalities.

Cognitive impairment occurs in approximately 47% of patients with primary-progressive multiple sclerosis (PP-MS)^1^, affecting a wide range of functions, from processing speed to executive functions and visuo-spatial memory^1^,^2^,^3^,^4^,^5^. Unlike the most common relapsing-remitting (RR) form of the disease, PP-MS is characterized by absence of relapses prior to clinical deterioration and by spinal cord focal and diffuse damage in presence of only few macroscopic inflammatory brain lesions^6^. This raises the question of what pathophysiological mechanisms underpin the development of cognitive impairment in PP-MS^7^. A recent study has suggested that it is related to the disruption of brain functional connectivity of the default mode network (DMN) as measured by resting-state functional MRI (rs-fMRI) and associated with microstructural abnormalities of the corpus callosum and cingulum^8^. However, while the relationship between cognitive-related functional connectivity abnormalities and the focal and diffuse damage of the connecting white matter (WM) tracts is well established in early and RR-MS^9^,^10^, it is not clear whether this holds true in PP-MS patients who are characterized by a relatively moderate WM lesion burden^6^ and a high burden of cortical lesions (CLs)^11^. Therefore, we postulated that, in addition to normal-appearing WM brain tissue injury, the presence of CLs might play a role in the modulation of functional connectivity in PP-MS patients.

Recent advances in rs-fMRI analysis have shown that the investigation of functional connectivity in multiple frequency bands and the assessment of blood-oxygen-level dependent (BOLD) signal amplitude variability could provide a more comprehensive characterization of brain functional correlates of cognitive impairment. Indeed, while functional connectivity of slow frequency bands (Slow-5: 0.01–0.027 Hz and Slow-4: 0.027–0.073 Hz)^12^,^13^ may contribute to the characterization of deep gray matter (GM) and midline cortical regions^14^,^15^,^16^, variability of BOLD signal amplitude across time might represent a direct index of neuronal activity. Several studies have shown that GM exhibits a higher BOLD signal amplitude than WM^14^,^17^ and that actively firing, highly connected neurons show a high spiking variability^18^. Thus suggesting that BOLD signal amplitude variation may reflect the variable metabolic demand of active neurons. Therefore, our aims were to: (i) identify the major domains of cognitive impairment in PP-MS through a principal component analysis (PCA) of neuropsychological tests and select resting state networks (RSNs) of interest accordingly; (ii) characterize, for the selected networks, functional connectivity and variability abnormalities associated with cognitive impairment in different frequency bands and, (iii) investigate whether the presence of CLs, which show a high prevalence in PP-MS patients^11^,^19^, has an impact on specific functional connectivity and variability changes.

## Methods

### Subjects

Twenty-five patients with PP-MS (mean age ± SD: 51.0 ± 9.8, range: 34–63 years) were consecutively enrolled from January 1, 2012 through December 31, 2013. Inclusion criteria were: (a) age between 25 and 65 years; (b) diagnosis of clinically definite MS, according to the revised McDonald criteria^20^ and a PP course; (c) Expanded Disability Status Scale (EDSS) score no greater than 6.5 at screening; (d) if treated, patients had to be on stable treatment for at least 1 year. Twenty gender- and age-matched healthy volunteers (mean age ± SD: 50.9 ± 10.5, range: 32–65 years) were recruited as controls (CTRLs). For all participants, the following exclusion criteria were applied: (a) current or past history of major hematological, renal, hepatic, psychiatric disorder or neurologic disease other than MS (for the patient group); (b) contraindications to MRI. On the same day of MRI acquisition patients underwent neurological evaluation with EDSS rating.

The institutional review board of Icahn School of Medicine at Mount Sinai approved the study and all participants gave written informed consent before investigation according to the Declaration of Helsinki. All methods were performed in accordance with the relevant guidelines and regulations.

### Neuropsychological assessment and analysis

Neuropsychological evaluation was performed on the same day of the MRI session, administering the minimal assessment of cognitive function in MS (MACFIMS)^21^. Raw scores from MACFIMS battery were converted in z-scores^22^. The MACFIMS battery includes tests exploring sustained attention, concentration, processing speed and working memory (paced auditory serial addition test at 3 s –PASAT; symbol digit modalities test -SDMT), verbal and visuo-spatial episodic memory (California verbal learning test II-CVLT-II; brief visuo-spatial memory test revised - BVMT-R), executive functions (Delis-Kaplan executive function scale sorting test -DKEFS; controlled oral word association test -COWAT) and spatial processing (judgment of line orientation test-JLO). Six CTRLs were non-native English speakers and did not perform MACFIMS evaluation. Raw scores were converted in z-scores. A PCA of the neuropsychogical tests (including both patients and controls) was performed using SPSS (version 20.0, IBM, Chicago, Illinois) and the first component’s eigenvalues were used to calculate a weighted average of the z-scores of the neuropsychological tests to obtain a global cognitive (GC) score. Patients were divided into cognitively impaired and cognitively preserved using a hierarchical clustering analysis of the GC score using Ward’s method with squared euclidian distance.

### MRI acquisition

Participants underwent MRI on a 3.0 T scanner (Achieva, Philips, The Netherlands). The MRI protocol included: (a) axial dual echo (DE) turbo spin echo (TSE) sequence: repetition time (TR) = 2500 ms, echo time (TE1) = 10 ms, TE2 = 80 ms, field of view (FoV) = 230 × 230 mm^2^, matrix size = 512 × 512, 46 contiguous 3 mm-thick slices; (b) sagittal 3D T1-weighted turbo field echo (TFE) sequence: TR = 7.5 ms, TE = 3.5 ms, inversion time (TI) = 900 ms, flip angle = 8°, voxel size = 1 × 1 × 1 mm^3^, 172 contiguous slices; (c) axial phase-sensitive inversion recovery (PSIR) sequence: TR = 4500 ms, TE = 8 ms, TI = 400 ms, 46 contiguous 3 mm thick slices with in-plane reconstructed resolution of 0.5 × 0.5 mm^2^; (d) T2* echo-planar imaging (EPI) for resting state fMRI: TR = 2300 ms, TE = 27 ms, 150 volumes; voxel size = 3 × 3 × 3 mm^3^. During resting state fMRI acquisition, subjects were asked to rest with their eyes closed without falling asleep; (f) a single-shot EPI sequence for diffusion tensor imaging: TR = 8550 ms, TE = 89.5 ms, voxel size = 2 × 2 × 2 mm^3^; b-values = 0, 1000, 2000 s/mm^2^ and 32 directions each.

### Structural MRI analysis

Brain WM T2 and T1 lesion volumes (LV) were measured as previously described in Ghassemi and colleagues^23^. CLs were identified and segmented by a single observer blinded to clinical information and subject’s identity, under the supervision of a senior investigator, according to published consensus criteria^24^ on PSIR images (Jim 6; Xinapse Systems, England), and classified as intracortical (IC), leucocortical (LC) or juxtacortical (JC)^24^. JC lesions, involving only WM, were excluded from further analysis. T2 and CL probability maps were created by creating a mean image of lesion maps (sum of binarized individual maps divided by number of patients) after registration to common spatial template (see below).

Maps of GM z-scores used for subsequent fMRI analysis were calculated as described in Villain and colleagues^25^. Briefly, the T1-weighted images of all study subjects were segmented into GM, WM and cerebro-spinal fluid (CSF) maps with FSL VBM (http://fsl.fmrib.ox.ac.uk/fsl/fslwiki/FSLVBM). The GM maps were then registered to the GM population-specific template generated from the complete image set masked with the GM population template (thresholded in order to retain only pixels having probability >30% to belong to the GM). The resulting individual GM maps were then masked with the thresholded GM population template and used to create z- score maps of GM atrophy by applying the following formula: ([subject individual GM value -control mean GM value]/control [SD] GM value). Individual z- score maps of GM atrophy and conservative GM masks, created for subsequent variability analysis by thresholding the individual GM probability maps at 70%, were finally normalized to the MNI space.

Tract-based Spatial Statistics (TBSS) was performed to compare WM microstructural damage between cognitively preserved and impaired patients. The b0 maps were used to extract the brain by using BET from FSL (www.fmrib.ox.ac.uk/fsl/). The diffusion images were co-registered and the Diffusional Kurtosis Estimator (DKE) software^26^ was used to obtain fractional anisotropy (FA) maps. Then, using the TBSS pipeline, individual subjects’ FA maps were registered to a common space (FMRIB58_FA standard-space) using non-linear registration with FNIRT. A mean FA skeleton was created representing the centers of all tracts common to all the subjects and then each subject’s individual aligned FA map was projected onto the skeleton.

### Resting state fMRI data preprocessing

Functional preprocessing steps were implemented in Analysis of Functional NeuroImages (AFNI)^27^ (http://afni.nimh.nih.gov/afni) and FSL. First, removal of the first 3 volumes of each functional dataset and slice-timing correction were performed using AFNI, followed by spatial smoothing (Gaussian kernel, FWHM = 6 mm) and removal of motion artifacts with FSL and ICA-AROMA^28^,^29^. In AFNI, T1-weighted and functional images for all subjects were then were normalized to the 2 × 2 × 2 mm^3^ MNI template. The mean time-series from the WM and the CSF were regressed out. The data were then bandpass-filtered between 0.01 and 0.1 Hz, the standard frequency band (SFB) thought to reflect mainly neuronal fluctuations^30^. Based on recent findings in healthy subjects^12^,^14^, we also focused on two separate frequency sub-bands within the SFB: Slow-5 (0.01–0.027 Hz) and Slow-4 (0.027–0.073 Hz).

### Functional connectivity and variability

A seed-based analysis of resting state functional connectivity was conducted using AFNI. Spheres of 6 mm radius were used as seeds in regions of interest (ROI) in the dorsal attention network (DAN), right attentional network (RAN) and executive control network (ECN) according to published data^31^. The DAN, RAN and ECN were chosen as networks of interest corresponding to the cognitive domains found to be relevant in the PCA of the neuropsychological tests. Since the ECN, DAN and RAN show a better spatial differentiation in the frontal than in the parietal regions^31^,^32^, we decided to focus on the frontal and temporal areas in order to avoid the selection of partially overlapping seed ROIs. For the DAN, two seed-ROIs were selected in the left and right frontal eye fields (FEF) (MNI x, y, z coordinates −29,−5, 55, and 31,−5, 54); for the ECN, three seed-ROIs were used in the dorso-medial, left anterior and right anterior prefrontal cortex (dmPFC, laPFC, raPFC) (MNI x, y, z coordinates 1, 30, 44; −45, 50,−5; 46, 51,−7); for the RAN, two seed-ROIs were chosen in the right middle frontal gyrus and right middle temporal gyrus (rMFG, rMTG) (MNI x, y, z coordinates 34, 24, 44; 64, −39, −11). Mean time-series for each seed-ROI was calculated by averaging the time-series of all voxels within the sphere. Correlation analysis was performed between each seed-ROI and all other brain voxels for each frequency band (SFB, Slow-5 and Slow-4). Fisher’s r-to-z transform was used to obtain spatial z-maps with each voxel’s intensity representing its correlation strength with the seed-ROI.

Variability of the BOLD signal was measured by calculating the standard deviation of the time-series amplitude within the seed-ROIs, in each frequency band. Whole-brain variability was investigated by calculating the standard deviation within the entire GM^15^, using the conservative GM masks.

Results were anatomically labeled using the Talairach-Tournoux Atlas available in AFNI. Because head motion can affect BOLD time-series amplitude and is often higher in patient populations, we compared head motion (mean relative frame-to-frame displacement-FD calculated using FSL) between patients and CTRLs.

### Statistical analysis

Between-group differences of gender and treatment were assessed with Fisher’s exact Test and Chi-square Test, whereas differences in other demographic, clinical and head motion parameters were tested with Mann-Whitney U Test and Kruskal-Wallis Test using the SPSS software package (version 20.0, IBM, Chicago, Illinois). Between-group differences in MRI structural parameters were tested with a multivariate analysis of variance, entering age as a covariate. Statistical significance was set at p < 0.006, Bonferroni corrected for multiple comparisons.

TBSS voxel-wise non-parametric statistics were performed to compare FA spatial maps between cognitively impaired and cognitively preserved groups, using *randomise* with 5000 permutations, entering age as a covariate of no interest. Statistical significance was set at p < 0.05, Threshold-Free Cluster Enhancement corrected for multiple comparisons.

To compare functional connectivity between patients and CTRLs, voxel-wise statistics were run in AFNI, using a two-sample t-test in each frequency band. To exclude a possible influence of regional GM atrophy on the voxel-wise comparisons of resting state activity between CTRLs and PP-MS patients, the z-scores of GM volume were used as a confounding voxel-wise covariate in the statistical analysis as described previously^25^. Age was also used as covariate to control for potential aging effects, as well as FD, in order to take into account the effects of head motion. In patients, voxel-wise correlations between CL/IC lesions volume and the functional connectivity maps showing significant differences in the between group comparisons were performed in AFNI, entering age, GM z-scores and FD as covariates. For both analyses, the resulting t-maps were thresholded at a corrected p-value of 0.01 (multiple-comparisons corrected using the Monte Carlo simulation as implemented in Alphasim, AFNI).

Mean variability values extracted from GM and seed regions were compared in SPSS, using an ANOVA corrected for age, GM volume and FD, Bonferroni corrected at p < 0.002.

In patients, voxel-wise correlations between CL/IC lesions volume and variability in each frequency band were performed in AFNI, entering age, GM z-scores and FD as covariates and thresholding the resulting t-maps at a corrected p value of 0.01 (multiple-comparisons corrected using the Monte Carlo simulation as implemented in Alphasim, AFNI).

In patients, voxel-wise correlations between functional connectivity and variability in GM were performed in AFNI, entering age, GM z-scores and FD as covariates. All resulting t-maps were thresholded at a corrected p value of 0.01 (multiple-comparisons corrected using the Monte Carlo simulation as implemented in Alphasim, AFNI).

## Results

### Clinico-psyhcological features and structural MRI

Table 1 summarizes the main clinical and structural MRI characteristics of the participants. An age distribution plot is provided in Supplementary Material (Supplementary Fig. 1).

**Figure 1:**
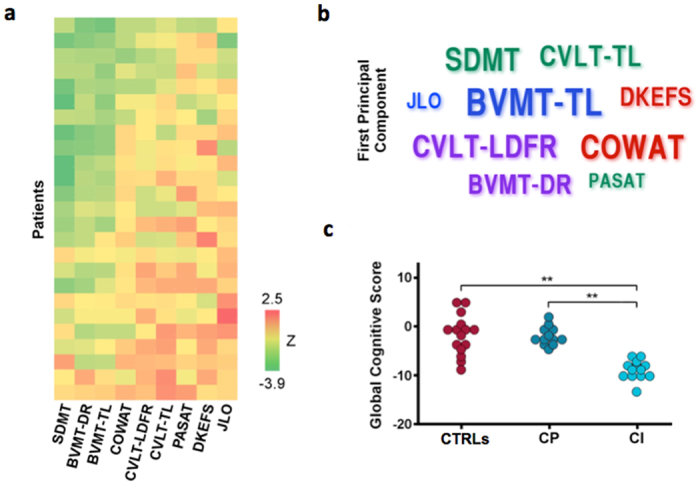
Cognitive status of PP-MS patients. Panel (a) shows results of the neuropsychological tests for all patients (z-score). Rows represent individual patients and columns each test, both sorted by increasing mean z-score, indicated by the color code. Panel (b) presents the neuropsychological tests used in the PCA analysis, with each name scaled by the loadings of the first principal component for the patient dataset. Colors indicate cognitive domain: executive functions (red), visuo-spatial ability (blue), short-term memory (purple) and working memory/processing speed (green). Panel (c) shows the global cognitive scores for healthy controls (CTRLs), cognitively impaired (CI) and cognitively preserved (CP) patients. Asterisks represent level of significance for Mann-Whitney test (p < 0.001). Abbreviations: SDMT = symbol digit modalities test, BVMT-DR = brief visuo-spatial memory test-delayed recall, BVMT-TL = brief visuo-spatial memory test-total learning, COWAT = controlled oral word association test, CVLT-LDFR = California verbal learning test-long delayed free recall, CVLT-TL = California verbal learning test-total learning, PASAT = paced auditory serial addition test at 3 s, DKEFS = Delis-Kaplan executive function scale sorting test, JLO = judgment of line orientation test.

PP-MS patients showed a wide range of cognitive deficits (Fig. 1a). PCA of the neuropsychological tests yielded a first principal component that explained 40.26% of the variance. The main tests contributing to this component were the BVMT-R total learning (weight 0.828) and the COWAT (weight 0.727) (Fig. 1b). Therefore, our subsequent functional analysis was focused on the DAN, RAN and ECN. The DAN, involved in spatial orientation of attention^33^ and visuospatial working memory^34^,^35^,^36^, is composed of the intraparietal sulcus and the junction of the precentral and superior frontal sulcus (frontal eye field, FEF) in each hemisphere. The RAN, engaged in attention reorientation^37^ and visuospatial processing^38^, is composed of the temporal-parietal junction and ventral frontal cortex^33^. The ECN facilitates higher cognitive processes including flexibility of behavioral choices in response to shifting conditions and context, and includes known sites for sustained attention/working memory (dorsolateral-prefrontal cortex, lateral parietal cortex)^39^ and response selection (dorsomedial-frontal cortex)^40^.

The first component’s eigenvectors were used to calculate a weighted average of the z-scores of the neuropsychological tests to obtain a GC score. Based on the clustering analysis, 13 patients were classified as cognitively impaired and 12 patients as cognitively preserved. Significant differences in GC score were identified between cognitively impaired and preserved patients (−8.94 ± 2.00 vs −1.90 ± 1.88, p = 0.000022) and between cognitively impaired patients and HC (−8.94 ± 2.00 vs −1.80 ± 4.09, p = 0.000046) (Fig. 1c).

Controls, cognitively preserved, and cognitively impaired MS patients were matched for gender, and level of education. While no differences in terms of disease modifying treatment, disease duration, EDSS and WM lesion loads were detected between cognitively preserved and impaired patients, cognitively preserved patients resulted significantly older (p = 0.030). Cognitively impaired patients showed a significantly higher IC lesion volume (respectively p = 0.004). Comparison of cognitively preserved and impaired patients WM microstructural damage showed a widespread significant decrease in FA in the cognitively impaired group (Supplementary Fig. 2a). WM and GM lesion distribution is shown in Supplementary Fig. 2b.

### Head motion analysis

No significant differences were detected between PP-MS patients and CTRLs (0.25 ± 0.20 vs 0.19 ± 0.10, p = 0.479) and between cognitively preserved and impaired patients in terms of FD (0.29 ± 0.25 vs 0.21 ± 0.11, p = 0.894).

### Resting state fMRI FC analysis

The regional differences in functional connectivity between CTRLs and PP-MS patients for the DAN, RAN and the ECN seed regions, in the three frequency bands are reported in detail in Supplementary Table 1 (p = 0.01, Monte Carlo corrected for multiple-comparisons).

### CTRLs vs PP-MS patients

**Figure 2:**
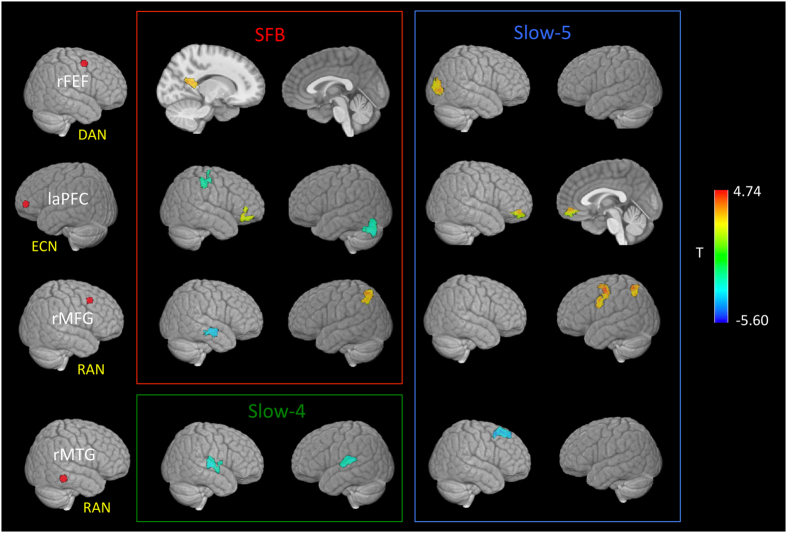
Clusters showing a significant difference in FC between PP-MS patients and healthy controls. Seed regions (first column) for the dorsal attention network-DAN (right frontal eye field-rFEF), executive control network-ECN (lateral anterior prefrontal cortex-laPFC) and right attentional network-RAN (right middle frontal gyrus-rMFG, right middle temporal gyrus-rMTG) are shown. Significant results are shown for the standard frequency band (SFB; 0.01–0.1 Hz), Slow-5 (0.01–0.027 Hz) and Slow-4 (0.027–0.073 Hz). All t-maps are thresholded at corrected p < 0.01 (covariates: age, grey matter z-scores and mean relative frame-to-frame displacement). The color bar shows voxel-wise T-values.

In the SFB (Fig. 2, red panel), compared to CTRLs, PP-MS patients showed increased connectivity between the frontal DAN and regions of the DMN and between the fronto-parietal regions of the ECN and frontal areas of the RAN. Reduced connectivity was detected between the frontal ECN, cerebellum and primary somatosensory cortex.

In Slow-5 (Fig. 2, blue panel), PP-MS patients showed increased connectivity between: the frontal DAN and temporal areas of the RAN; the frontal ECN and the DMN; the frontal RAN and the frontal region of the left attentional network (LAN) and the parietal ECN component. Decreased connectivity was found between the temporal RAN component and the supplementary motor area.

In Slow-4 (Fig. 2, green panel), patients showed decreased connectivity between the temporal RAN region and the insular node of the salience network-SN. In both SFB and Slow-4 within network disconnection was present in the RAN (Fig. 2).

### CTRLs vs cognitively impaired patients

**Figure 3:**
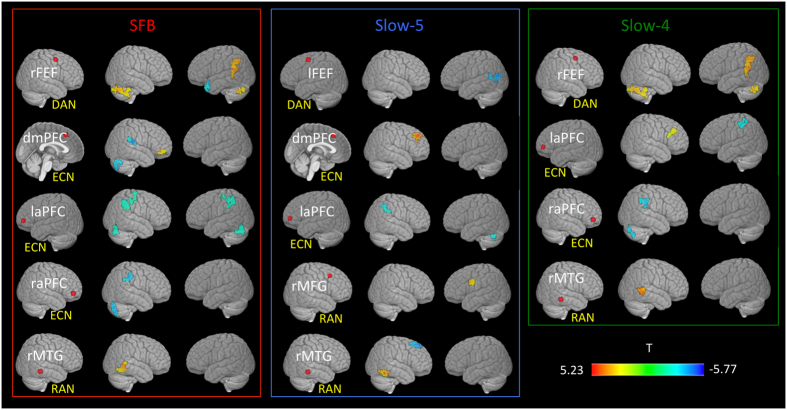
Clusters showing a significant difference in FC between cognitively impaired patients and controls. Seed regions for the dorsal attention network-DAN (right frontal eye field-rFEF, left frontal eye field-lFEF), executive control network-ECN (dorsomedial prefrontal cortex-dmPFC, left anterior prefrontal cortex- laPFC, right anterior prefrontal cortex-raPFC), and right attentional network-RAN (right middle temporal gyrus-rMTG, right middle frontal gyrus-rMFG) are shown as red dots. Significant results are reported for frequency band the standard frequency band (SFB; 0.01–0.1 Hz), Slow-5 (0.01–0.027 Hz) and Slow-4 (0.027–0.073 Hz). All t-maps are thresholded at corrected p < 0.01 (covariates: age, grey matter z-scores and mean relative frame-to-frame displacement). The color bar shows voxel-wise T-values.

In the SFB (Fig. 3, red panel), patients showed decreased connectivity between the frontal ECN components, the insula and parietal RAN regions. Reduced connectivity was also present between the frontal DAN and the temporal RAN component.

In Slow-5 (Fig. 3, blue panel), patients showed increased connectivity of both the fronto-temporal RAN and the fronto-medial ECN components with the DMN. They also showed decreased connectivity between the frontal DAN and parietal LAN areas, between the frontal ECN and parietal RAN areas, and between the temporal RAN and the supplementary motor area.

In both SFB and Slow-4 (Fig. 3, green panel), patients showed increased connectivity between the frontal DAN and the cerebellum, between the frontal DAN and the cingulate gyrus, between the frontal ECN and the RAN, and within temporal components of the RAN. They also showed decreased connectivity between the frontal ECN and the primary somatosensory cortex.

Across all frequency bands, decreased connectivity was detected between the frontal ECN components and the cerebellum.

### CTRLs vs cognitively preserved patients

**Figure 4:**
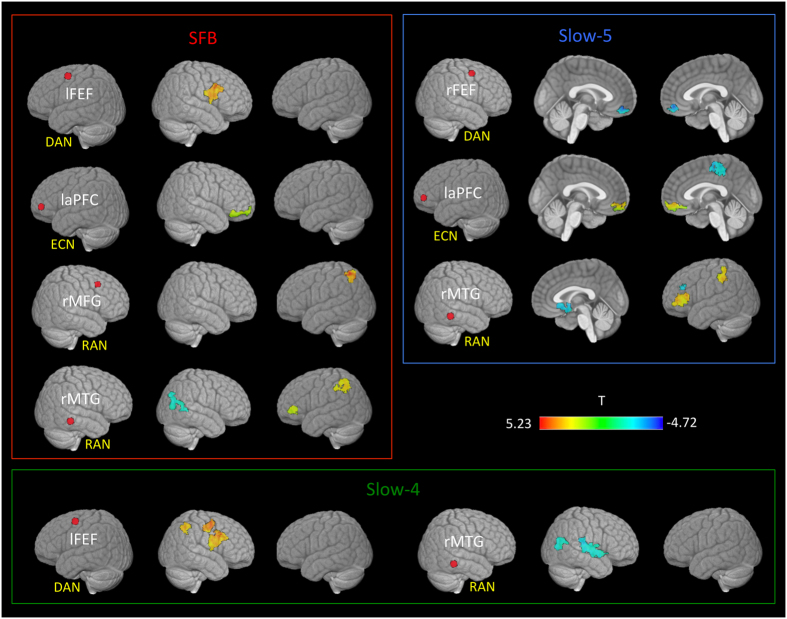
Clusters showing a significant difference in FC between cognitively preserved patients and controls. Seed regions for the dorsal attention network-DAN (right frontal eye field-rFEF, left frontal eye field-lFEF), executive control network-ECN (left anterior prefrontal cortex- laPFC), and right attentional network-RAN (right middle temporal gyrus-rMTG, right middle frontal gyrus-rMFG) are shown as red dots. Significant results are reported for the standard frequency band (SFB; 0.01–0.1 Hz), Slow-5 (0.01–0.027 Hz) and Slow-4 (0.027–0.073 Hz). All t-maps are thresholded at corrected p < 0.01 (covariates: age, grey matter z-scores and mean relative frame-to-frame displacement). The color bar shows voxel-wise T-values.

Compared to CTRLs, in the SFB (Fig. 4, red panel), patients showed increased connectivity between the temporal RAN and the frontal LAN components and within the ECN. Decreased connectivity was present between the temporal RAN region and the DMN.

In Slow-5 (Fig. 4, blue panel), patients showed decreased connectivity between the frontal components of DAN and the fronto-medial DMN, between the frontal components of ECN and the primary motor cortex and between the temporal components of RAN and the cingulate components of SN. They also showed increased connectivity between the frontal components of ECN and the fronto-medial DMN and between temporal areas of RAN, the frontal component of DMN and parietal component of LAN.

In Slow-4 (Fig. 4, green panel) patients showed decreased connectivity between the temporal component of RAN and occipital areas and within temporal components of RAN and increased connectivity between the frontal components of DAN and ECN.

In both the SFB and Slow-4, patients showed increased FC between the frontal components of the DAN and RAN.

### Cognitively preserved vs cognitively impaired patients

**Figure 5:**
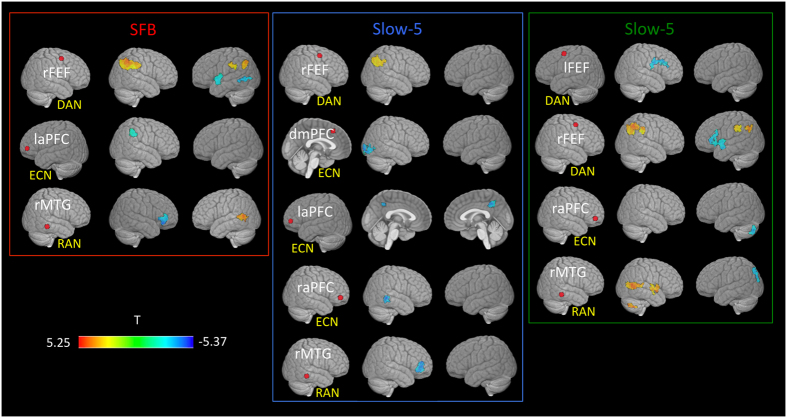
Clusters showing a significant difference in FC between cognitively impaired and cognitively preserved patients. Seed regions for the dorsal attention network-DAN (right frontal eye field-rFEF, left frontal eye field-lFEF), executive control network-ECN (dorsomedial prefrontal cortex-dmPFC, left anterior prefrontal cortex-laPFC, right anterior prefrontal cortex-raPFC), and right attentional network-RAN (right middle temporal gyrus-rMTG) are shown as red dots. Significant results are reported for the standard frequency band (SFB; 0.01–0.1 Hz), Slow-5 (0.01–0.027 Hz) and Slow-4 (0.027–0.073 Hz). All t-maps are thresholded at corrected p < 0.01 (covariates: age, grey matter z-scores and mean relative frame-to-frame displacement). The color bar shows voxel-wise T-values.

The direct comparison of cognitively impaired and preserved patients showed, in the SFB (Fig. 5, red panel) decreased connectivity between the frontal DAN component, the insula and occipital areas.

In Slow-5 (Fig. 5, blue panel), cognitively impaired patients showed decreased FC between frontal ECN components and the temporal RAN, cingulate and occipital cortex.

In Slow-4 (Fig. 5, green panel), cognitively impaired patients showed decreased connectivity between the frontal DAN, the fronto-temporal DMN and temporal RAN regions, between frontal ECN and the cerebellum, and between the temporal and parietal regions of the RAN. They also showed increased connectivity between frontal DAN components and the cingulate cortex, and between the temporal RAN and the cerebellum.

In the SFB and Slow-5, cognitively impaired patients showed decreased connectivity within temporal and frontal components of the RAN, while in the SFB and Slow-4 increased connectivity was present within the temporal regions of the RAN.

Across all frequency bands cognitively impaired patients showed increased connectivity between the frontal components of DAN and the parietal component of RAN.

### Variability analysis

Between groups comparison of variability in GM (global variability) and seed ROIs (regional variability) showed constantly higher variability in patients than CTRLs and in cognitively impaired patients in comparison with cognitively preserved patients. Global variability was higher in PP-MS patients than CTRLs (SFB 0.43 vs 0.37; Slow-5 0.25 vs 0.20; Slow-4 0.30 vs 0.25, p < 0.0001) and in cognitively impaired in comparison with cognitively preserved (SFB 0.46 vs 0.41; Slow-5 0.27 vs 0.23; Slow-4 0.31 vs 0.28, p < 0.001). Significant results of regional variability analysis are reported in Table 2.

### Correlations between functional connectivity/variability and CL/IC lesions volume

Significant correlations with connectivity were found in several regions in all frequency bands for both CL and IC lesion volume (detailed results in Supplementary Table 2). Significant correlations with variability were found for CL volumes in the cingulate gyrus and superior frontal gyrus (detailed results in Supplementary Table 3) (p 0.01, Monte Carlo corrected for multiple-comparisons).

### Correlations between functional connectivity and variability

Significant correlations between connectivity and global variability was found in several regions in all frequency bands (p 0.01, Monte Carlo corrected for multiple-comparisons). The detailed results are reported in Supplementary Table 4.

## Discussion

Using a data-driven approach, we identified visuo-spatial memory and executive functions as the most impaired cognitive domains in our patients, confirming previous findings in PP-MS^4^,^5^.

The functional connectivity analysis of the DAN, RAN and ECN confirmed the presence of a widespread cortical reorganization, involving the connection between areas pertaining to these networks and regions involved in other networks also responsible for cognitive control (i.e. LAN, DMN and SN). Such functional reorganization mainly resulted in increased between-network connectivity, with hyper-synchronization of frontal attentional networks (RAN-LAN), frontal and dorsal attentional systems (RAN-DAN), executive and attentional networks (ECN-RAN) and connections to the DMN (DAN-DMN, ECN-DMN). Disconnection resulted limited to RAN within network connectivity, connections between RAN and SN as well as connections between ECN/RAN and areas involved in motor control.

The specificity of our results in different frequency bands confirms the higher sensitivity of the lowest bands to fluctuations occurring along the midline structures (Slow-5)^15^,^16^, thus supporting the concept that resting-state BOLD oscillations exhibit a frequency-dependent, anatomically constrained spatial structure^13^.

When the patients were stratified on the basis of cognitive impairment and compared with CTRLs, our results disclosed the presence of a large-scale reconfiguration of brain networks interaction, with different characteristics in cognitively impaired and preserved patients. In cognitively preserved patients increased synchronicity was present between attentional networks (DAN, RAN, LAN), and within ECN. In cognitively impaired patients the connections between networks involved in the control of macro-domains (DAN, RAN and LAN for attention; ECN, SN, motor areas for stimulus selection, planning and integration) showed reduced synchronicity, with hyper-synchronicity being limited to the connections between networks functionally more segregated (ECN-RAN) and between attentional networks and cerebellum.

Functional abnormalities between large-scale neuronal networks and within DMN sub-regions have been described in RR-MS^41^,^42^ with cognitive impairment. In one study, RR-MS patients showed increased connectivity between the ECN and SN and decreased connectivity between the ECN and DMN when compared to healthy CTRLs^42^. In a second study, RR-MS patients without cognitive impairment showed a weaker functional connectivity in the anterior cingulate cortex and in the core of the posterior cingulate cortex (PCC), and a stronger connectivity at the periphery of the PCC^41^. In PP-MS patients, rather than an intra-network adaptation, we found a differential inter-network reorganization of DMN regions. Specifically, cognitively preserved patients showed increased synchronicity between both RAN and ECN and the frontal component of DMN and reduced synchronicity between temporal RAN and posterior cingulate cortex, as well as between frontal areas of DAN and DMN while cognitively impaired patients showed increased synchronicity between fronto-temporal areas of the attentional network (RAN) and fronto-temporal DMN, between frontal areas of DAN and cingulate cortex as well as between frontal ECN and frontal DMN.

Although we cannot infer from our results the beneficial vs maladaptive role of such functional reorganization^43^, the loss, in cognitively impaired patients, of the hyper-synchronicity between RSN involved in strictly related functions and the differential reorganization between these networks and the DMN, whose central role in cognitive systems integration is well known^44^, seems to suggest that these rearrangements could be responsible for the relative functional preservation observed in cognitively preserved patients.

Interestingly, in addition to the different functional connectivity reorganization, cognitively impaired patients showed higher prevalence of IC lesions and more widespread WM injury when compared with cognitively preserved patients. The disruption of physiological connections determined by diffuse WM damage and focal GM lesions in PP-MS could result in the loss of some connections and in the amplification of the connections still structurally preserved, without necessarily determining any functional advantage in terms of cognitive ability. Since in our patients the functional connectivity rearrangement was not associated with the maintenance of an adequate cognitive status, we believe it may suggest the presence of maladaptive reorganization, possibly related to the extent of WM structural damage and the presence of CLs^45^. Indeed, we found significant correlations between the number of CLs and functional connectivity, suggesting that the presence of lesions in the brain cortex might indeed have a role in the functional reorganization of brain networks.

Perhaps, the most novel aspect of our study is related to the variability analysis that revealed increased values in both the whole group of patients and in the two subgroups when compared with CTRLs, and in cognitively impaired when compared with cognitively preserved patients. Variability is considered an index of neural activity^46^,^47^. It increases with brain maturation^47^ and decreases with age showing a bidirectional pattern^46^. The existence of a spatial pattern, and a fluid transition of variability status across life stages seem to be a critical index of the cognitive capacity^46^. Although it remains speculative, the increased variability in PP-MS could reflect a dysfunctional neural activity responsible for the disruption of networks interactions. In particular, previous research suggests that there is an optimal level of variability that facilitates neural function^46^,^48^; it is therefore possible that a homogeneous increase in neuronal variability would affect the functional integration of brain networks. In alternative, the increased variability could represent a failed attempt to compensate for GM lesional damage, as suggested by the presence of a direct correlation between CLs volume and variability. In this scenario, the abnormal network integration would elicit an enhanced neuronal activity in structurally preserved GM areas.

Theoretically, variability should be independent of functional connectivity^49^, but recent studies have reported a close relationship between them^50^,^51^. While in healthy subjects higher variability correlates with stronger functional connectivity^51^, in presence of brain pathology this correlation is lost^15^. In our population, the two metrics showed a widespread voxelwise correlation. In patients, variability showed both direct and inverse correlations with functional connectivity in different brain areas, suggesting that a maladaptive increase in neural activity might be associated with the reorganization of functional connectivity. Our study is not without limitations. The small sample size may have limited our ability in determining significant differences in the subgroup comparisons. Most of our patients were under treatment with FDA approved disease-modifying agents; while we cannot rule out a potential treatment effect on measures of brain functional connectivity and variability or on the expression of cognitive deficits^52^, the lack of difference in type and frequency of treatment between the cognitively preserved and impaired patients should have minimized any confounding effects. Longitudinal studies on larger cohorts are needed to fully elucidate the role of the brain functional reorganization underlying the cognitive deficits in PP-MS and to differentiate the maladaptive from the compensatory modifications.

In conclusion, our results show for the first time that cognitive impairment in PP-MS patients is associated with increased variability and hypo-synchronicity of functionally-related/hyper-synchronicity of functionally-segregated large scale networks, which are, in turn, associated with CLs. While the variability analysis provides information on the neural activity underlying the functional connectivity abnormalities, the additional investigation of sub-frequency bands allows wider detection of functional abnormalities, with increased sensitivity along the midline structures. Hence, our novel findings contribute to improving characterization of the resting state correlates of cognitive impairment in PP-MS, and, once replicated in a larger cohort, may eventually be of use as a biomarker in clinical assessment and treatment.

## Additional Information

**How to cite this article:** Petracca, M. *et al*. Synchronization and variability imbalance underlie cognitive impairment in primary-progressive multiple sclerosis. *Sci. Rep.* **7**, 46411; doi: 10.1038/srep46411 (2017).

**Publisher's note:** Springer Nature remains neutral with regard to jurisdictional claims in published maps and institutional affiliations.

## Supplementary Material

Supplementary Information

## Figures and Tables

**Table 1:** Demographic and clinical characteristics of patients and controls.

	CTRLs	PP-MS	CP	CI
M/F^†^	9/11	12/13	4/8	8/5
Age, years (range)^‡^	51.0 ± 9.8 (34–63)	50.9 ± 10.5 (32–65)	55.7 ± 7.0 (45–65)	46.5 ± 11.5 (32–64)^*^
Education, years (range)^‡^	16.6 ± 2.4 (14–20)	16.0 ± 3.2 (11–22)	16.3 ± 3.0 (12–20)	15.7 ± 3.5 (11–22)
DMT^†^, count (percentage)	—	12 (48%)	4 (33%)	8 (61%)
Disease duration, years^‡^	—	8.0 ± 4.78	9.7 ± 4.8	8.1 ± 4.8
Median EDSS (range)^‡^	—	4.0 (1.5–6.0)	3.7 (2.0–6.0)	4.0 (1.5–6.0)
T2 LV, ml	—	5.76 ± 7.50	3.46 ± 4.85	7.89 ± 8.99
T1 LV, ml	—	3.07 ± 4.87	1.44 ± 1.97	4.57 ± 6.23
CL count	—	12.92 ± 11.88	8.64 ± 9.05	16.54 ± 13.09
CL LV, ml	—	0.67 ± 0.59	0.53 ± 0.55	0.79 ± 0.63
LC lesion count	—	7.92 ± 7.76	4.73 ± 6.02	10.61 ± 8.24
LC LV, ml	—	0.43 ± 0.44	0.31 ± 0.43	0.52 ± 0.43
IC lesion count	—	1.79 ± 2.00	0.54 ± 0.69	2.85 ± 2.15
IC LV, ml	—	0.05 ± 0.06	0.02 ± 0.02	0.09 ± 0.06^**^

Unless otherwise specified, all values are expressed as mean ± SD. *p < 0.05, **p < 0.006 (CP PP-MS vs CI PP-MS). ^†^Fisher’s exact Test/Chi-square Test. ^‡^Mann-Whitney U Test/Kruskal-Wallis Test. Abbreviations: CI = cognitively impaired, CL = cortical lesion, CP = cognitively preserved, CTRLs = controls, DMT = Disease modifying treatment, EDSS = expanded disability status scale, IC = intracortical, PP-MS = primary progressive multiple sclerosis, LC = leucocortical, LV = lesion volume.

**Table 2:** Between group comparison of variability in seed ROIs.

Frequency Band	Region	Mean Variability	
	Seed ROI	CTRLs	PP-MS
SFB	l FEF	0.26	0.31*
	r FEF	0.26	0.30*
	dmPFC	0.29	0.33
	l aPFC	0.54	0.64
	r aPFC	0.53	0.58
	r MFG	0.28	0.32
	r MTG	0.36	0.41*
Slow-5	l FEF	0.15	0.18*
	r FEF	0.13	0.16*
	dmPFC	0.16	0.18
	l aPFC	0.32	0.37
	r aPFC	0.32	0.35
	r MFG	0.16	0.19
	r MTG	0.18	0.24*
Slow-4	l FEF	0.18	0.21*
	r FEF	0.18	0.21*
	dmPFC	0.20	0.23*
	l aPFC	0.35	0.44
	r aPFC	0.35	0.39
	r MFG	0.19	0.22
	r MTG	0.25	0.28

*p < 0.002, Bonferroni corrected. Abbreviations: aPFC = anterior prefrontal cortex, CTRLs = healthy controls, dmPFC = dorso medial prefrontal cortex, FEF = frontal eye field, l = left, MFG = middle frontal gyrus, MTG = middle temporal gyrus, PP-MS = primary-progressive multiple sclerosis, r = right, ROI = region of interest, SFB = standard frequency band.
