# Supplementary material for: Synchronization and variability imbalance underlie cognitive impairment in primary-progressive multiple sclerosis
Source: Sci Rep. 2017 Apr 21;7:46411. doi: 10.1038/srep46411 (PMC5399449; doi:10.1038/srep46411)
Supplement: Supplementary Information [file srep46411-s1.doc]

**Synchronization and variability imbalance underlie cognitive impairment in primary-progressive multiple sclerosis**

M. Petracca, MD, PhD^; C. Saiote, PhD^; H.A. Bender, PhD; F. Arias, MS; C. Farrell, MA; P. Magioncalda, MD; M. Martino, MD; A. Miller, MD; G. Northoff, MD, PhD; F. Lublin, MD; M. Inglese*, MD, PhD

**Maria Petracca, MD, PhD** [maria.petracca@mssm.edu](mailto:maria.petracca@mssm.edu)

Department of Neurology, Icahn School of Medicine at Mount Sinai, NY, USA; Department of Neurosciences, Reproductive Sciences and Odontostomatology, University of Naples “Federico II”, Naples, Italy.

**Catarina Saiote, PhD** [catarina.saiote@mssm.edu](mailto:catarina.saiote@mssm.edu)

Department of Neurology, Icahn School of Medicine at Mount Sinai.

**Heidi A. Bender, PhD** [heidi.bender@mssm.edu](mailto:heidi.bender@mssm.edu)

Department of Neurology, Icahn School of Medicine at Mount Sinai.

**Franchesca Arias, MS** [arias.franchesca@gmail.com](mailto:arias.franchesca@gmail.com)

Department of Neurology, Icahn School of Medicine at Mount Sinai.

**Colleen Farrell, MA** [colleen.farrell@mssm.edu](mailto:colleen.farrell@mssm.edu)

Corinne Goldsmith Dickinson Center for MS Icahn School of Medicine at Mount Sinai, NY, USA

**Paola Magioncalda, MD** [paola.magioncalda@gmail.com](mailto:paola.magioncalda@gmail.com)

Department of Neuroscience, Rehabilitation, Ophthalmology, Genetics, Maternal and Child Health (DINOGMI), University of Genoa, Genoa, Italy

**Matteo Martino, MD** [matteomartino9@gmail.com](mailto:matteomartino9@gmail.com)

Department of Neuroscience, Rehabilitation, Ophthalmology, Genetics, Maternal and Child Health (DINOGMI), University of Genoa, Genoa, Italy

**Aaron Miller, MD** [aaron.miller@mssm.edu](mailto:aaron.miller@mssm.edu)

Corinne Goldsmith Dickinson Center for MS Icahn School of Medicine at Mount Sinai, NY, USA

**Georg Northoff, MD, PhD** [Georg.Northoff@theroyal.ca](mailto:Georg.Northoff@theroyal.ca)

Institute of Mental Health Research, University of Ottawa, Ottawa, Canada.

**Fred Lublin, MD** [fred.lublin@mssm.edu](mailto:fred.lublin@mssm.edu)

Corinne Goldsmith Dickinson Center for MS Icahn School of Medicine at Mount Sinai, NY, USA

**Matilde Inglese, MD, PhD** [matilde.inglese@mssm.edu](mailto:matilde.inglese@mssm.edu)

Department of Neurology, Radiology and Neuroscience Icahn School of Medicine at Mount Sinai; Department of Neuroscience, Rehabilitation, Ophthalmology, Genetics, Maternal and Child Health (DINOGMI), University of Genoa, Genoa, Italy

^These authors contributed equally to the manuscript.

*Corresponding author:

Matilde Inglese

Department of Neurology, Radiology and Neuroscience

Icahn School of Medicine at Mount Sinai

One Gustave L. Levy Place Box 1137, New York, NY 10029
Email: [matilde.inglese@mssm.edu](mailto:matilde.inglese@mssm.edu); Telephone: 212-824-9310; Fax: 212-348-1310

**Supplementary Fig.1.** Age distribution plot.


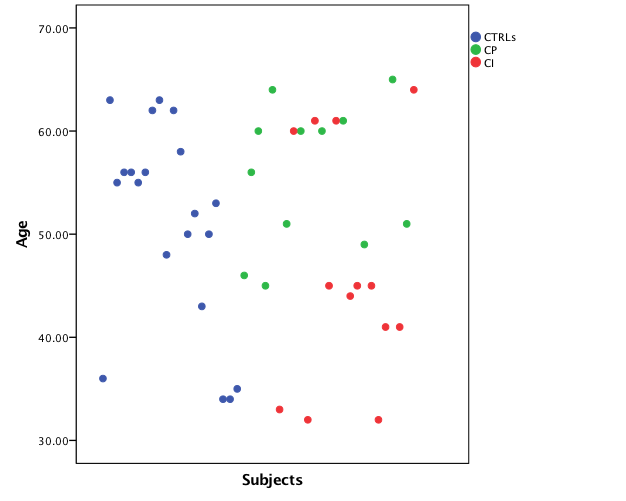


Healthy controls (CTRLs) are color coded in blue, cognitive preserved MS patients are color coded in green and cognitive impaired MS patients are color coded in red.

**Supplementary Fig.2** PP-MS structural damage.


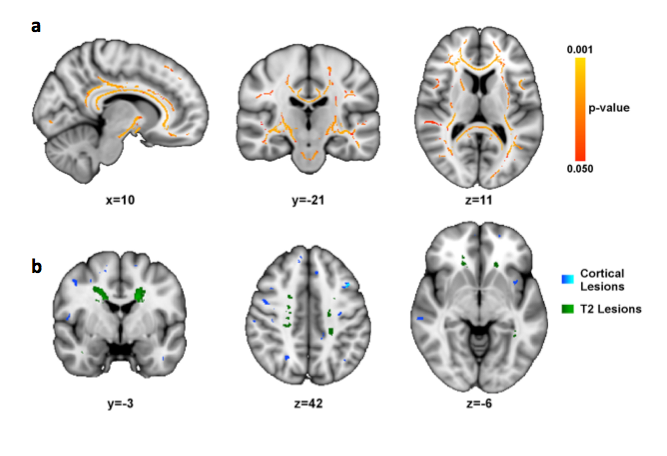


Panel **a** shows regions of significantly higher FA in cognitive preserved compared to cognitive impaired patients (two-sample t-test, age corrected), displayed on white matter skeleton derived using a Tract-based Spatial Statistics (TBSS) analysis. The color bar represents the p-value of significant results (p<0.05, Threshold-Free Cluster Enhancement corrected for multiple comparisons). Panel **b** shows cortical lesions (in blue) and white matter lesions (in green) probability maps (thresholded respectively between 0.04-0.08 and 0.1-0.4). Results are overlaid on MNI T1-weighted template (radiologic convention).

**Supplementary Table 1.** Between-group comparison of functional connectivity maps in standard frequency band (0.01-0.10 Hz), Slow-5 (0.01-0.027 Hz) and Slow-4 (0.027-0.073 Hz).

| **Groups** |  | **Seed ROI** | **Target Cluster** | **x, y, z MNI** | **Size** | **T** |
| --- | --- | --- | --- | --- | --- | --- |
| **CTRLs vs PP-MS** | **SFB** | r FEF | l PCG | -18,-44,16 | 281 | 3.96 |
|  |  | laPFC | l cerebellum^§^ | -2,-74,-20 | 524 | -5.60 |
|  |  |  | r MFG | 36,36,-14 | 274 | 3.35 |
|  |  |  | r PG^§^ | 24,-28,54 | 256 | -4.20 |
|  |  | r MFG | l SPL BA7 | -32,-56,58 | 338 | 3.74 |
|  |  |  | r MTG^§^ | 54,-4,-14 | 260 | -3.95 |
|  | **Slow-5** | r FEF | r MTG | 48,-80,14 | 283 | 4.74 |
|  |  | laPFC | r MedFG BA11 | 36,38.-14 | 354 | 3.48 |
|  |  |  | r IFG | 36,34,-18 | 214 | 4.11 |
|  |  | r MFG | l MFG | -42,-4,54 | 266 | 3.71 |
|  |  |  | l SPL BA7 | -24,-58,62 | 264 | 4.14 |
|  |  | r MTG | r SFG^§^ | 18,30,58 | 371 | -4.93 |
|  | **Slow-4** | r MTG | l insula BA13^§^ | -40,-24,16 | 346 | -4.73 |
|  |  |  | r STG^§^ BA6 | 46,-18,6 | 251 | -3.66 |
| **CTRLs vs CI** | **SFB** | r FEF | r cerebellum | 28,-78,-28 | 582 | 4.35 |
|  |  |  | l CG | -24,-51,20 | 361 | -4.67 |
|  |  |  | l STG^§^ | -40,18,-40 | 302 | -4.02 |
|  |  | dmPFC | r cerebellum^§^ | 46,-76,-32 | 361 | -5.74 |
|  |  |  | r insula BA13^§^ | 40,-36,20 | 358 | -4.11 |
|  |  |  | r MFG | 48,46,-14 | 279 | 4.69 |
|  |  | laPFC | r PC^§^ | 8,-44,48 | 1246 | -5.66 |
|  |  |  | l cerebellum^§^ | -2,-74,-20 | 691 | -5.77 |
|  |  |  | r PG^§^ | 24,-28,54 | 329 | -3.79 |
|  |  | raPFC | r cerebellum^§^ | 44,-74,-34 | 375 | -3.96 |
|  |  |  | r PG^§^ | 28,-30,42 | 269 | -3.64 |
|  |  | r MTG | r MTG | 64,-48,-6 | 384 | 4.33 |
|  | **Slow-5** | l FEF | l PC^§^ | -18,-76,18 | 344 | -4.44 |
|  |  | dmPFC | r MedFG | 14,40,38 | 286 | 4.11 |
|  |  | laPFC | r PC BA7^§^ | 14,-48,50 | 308 | -4.54 |
|  |  |  | l cerebellum^§^ | -34,-64,-30 | 236 | -4.18 |
|  |  | r MFG | l IFG | -50,12,26 | 262 | 3.20 |
|  |  | r MTG | r SFG^§^ BA6 | 16,40,52 | 363 | -4.30 |
|  |  |  | r ITG | 62,-48,-18 | 268 | 3.70 |
|  | **Slow-4** | r FEF | r cerebellum | 38,-50,-46 | 900 | 5.23 |
|  |  |  | l CG | -24,-51,22 | 860 | 4.32 |
|  |  |  | l cerebellum | -26,-66,-38 | 275 | 4.13 |
|  |  | laPFC | l PG BA3^§^ | -34,-34,56 | 372 | -4.26 |
|  |  |  | r MFG | 38,36,28 | 282 | 5.10 |
|  |  | raPFC | r cerebellum^§^ | 44,-75,-25 | 248 | -3.87 |
|  |  | r MTG | r MTG | 68,-44,-2 | 301 | 4.77 |
| **CTRLs vs CP** | **SFB** | l FEF | r MFG BA9 | 56,18,32 | 725 | 5.02 |
|  |  | laPFC | r SFG BA11 | 14,62,-16 | 349 | 4.95 |
|  |  | r MFG | l SPL BA7 | -36,-54,62 | 329 | 3.55 |
|  |  | r MTG | l IPL BA40 | -36,-54,48 | 456 | 3.52 |
|  |  |  | r PCG BA31^§^ | 22,-72,22 | 376 | -4.52 |
|  |  |  | l MFG | -40,44,0 | 258 | 4.39 |
|  | **Slow-5** | r FEF | l MedFG^§^ | -2,50,-16 | 348 | -3.09 |
|  |  | laPFC | r MedFG | 2,50,-12 | 454 | 4.23 |
|  |  |  | r PrecG BA4^§^ | 24,-28,56 | 427 | -4.03 |
|  |  | r MTG | l IFG | -38,30,10 | 510 | 4.28 |
|  |  |  | r SCG^§^ | 18,14,-12 | 287 | -4.66 |
|  |  |  | l ACG BA32^§^ | -2,38,22 | 262 | -3.76 |
|  |  |  | l IPL | -32,-32,42 | 241 | 3.85 |
|  | **Slow-4** | l FEF | r MFG BA9 | 56,18,32 | 889 | 5.18 |
|  |  |  | r MFG BA6 | 26,-6,48 | 314 | 4.00 |
|  |  |  | r SPL | 26,-54,48 | 280 | 5.23 |
|  |  | r MTG | r STG^§^ | 54,-20,6 | 908 | -3.51 |
|  |  |  | r cuneus^§^ | 24,-74,10 | 322 | -4.72 |
| **CP vs CI** | **SFB** | r FEF | r PC BA7 | 8,-68,42 | 1665 | 4.95 |
|  |  |  | r IPL BA40 | 50,-60,42 | 886 | 4.62 |
|  |  |  | l insula BA13^§^ | -36,2,-4 | 339 | -5.11 |
|  |  |  | l lingual G^§^ | -22,-66,0 | 265 | -3.86 |
|  |  | laPFC | r PC BA7^§^ | 4,-42,46 | 392 | -4.42 |
|  |  | r MTG | r STG | 52,-54,10 | 395 | 3.68 |
|  |  |  | l MFG BA11^§^ | -44,36,-14 | 342 | -4.03 |
|  | **Slow-5** | r FEF | r IPL | 48,-58,40 | 689 | 4.11 |
|  |  |  | r PC BA7 | 6,-70,46 | 414 | 5.25 |
|  |  | dmPFC | r cuneus^§^ | 16,-98,0 | 318 | -4.74 |
|  |  | laPFC | r CG BA31^§^ | 4,-42,44 | 303 | -4.33 |
|  |  | raPFC | r MTG^§^ | 70,-42,-6 | 241 | -5.09 |
|  |  | r MTG | r MFG^§^ | 44,48,-4 | 444 | -5.37 |
|  | **Slow-4** | l FEF | r IFG^§^ | 48,2,26 | 501 | -4.27 |
|  |  | r FEF | l STG BA38^§^ | -36,4,-14 | 688 | -4.63 |
|  |  |  | l PC BA7 | -14,-60,38 | 643 | 3.83 |
|  |  |  | r IPL | 56,-50,52 | 503 | 4.33 |
|  |  |  | r CG BA31 | 4,-30,42 | 394 | 4.49 |
|  |  |  | l uncus^§^ | -22,-4,-24 | 292 | -3.35 |
|  |  | raPFC | l cerebellum^§^ | -20,-80,-38 | 299 | -4.91 |
|  |  | r MTG | r STG | 64,-40,6 | 886 | 4.38 |
|  |  |  | r STG | 56,8,-10 | 309 | 3.92 |
|  |  |  | l PC BA7^§^ | -24,-76,52 | 252 | -4.38 |
|  |  |  | r cerebellum | 40,-70,-42 | 246 | 4.54 |

T-test adjusted for age, mean relative frame-to-frame displacement (FD) and GM z-scores, corrected for multiple comparisons at p<0.01. All reported clusters show increased connectivity with the seed ROI unless otherwise specified (^§^=clusters showing decreased connectivity with the seed region). Abbreviations: ACG=anterior cingulate gyrus, BA=Broadmann area, CI=cognitively impaired, CG=cingulate gyrus, CP=cognitively preserved, CTRLs=controls, dmPFC=dorso medial prefrontal cortex, FEF=frontal eye field, IFG=inferior frontal gyrus, IPL=inferior parietal lobule, ITG=inferior temporal gyrus, l= left, laPFC=left anterior prefrontal cortex, MedFG=medial frontal gyrus, MFG=middle frontal gyrus, MTG=middle temporal gyrus, PC=precuneus, PCG=posterior cingulate gyrus, PG=postcentral gyrus, PP-MS=primary-progressive multiple sclerosis, PrecG=precentral gyrus, r= right, raPFC=right anterior prefrontal cortex, SCG=subcallosal gyrus, SFG=superior frontal gyrus, SPL=superior parietal lobule, STG=superior temporal gyrus.

**Supplementary Table 2.** Correlation between voxelwise functional connectivity and CL or IC lesion volumes in the standard frequency band (0.01-0.10 Hz), Slow-5 (0.01-0.027 Hz) and Slow-4 (0.027-0.073 Hz).

| **Lesions** |  | **Seed ROI** | **Target Cluster** | **x, y, z MNI** | **Size** | **T** |
| --- | --- | --- | --- | --- | --- | --- |
| **CLs** | **SFB** | r FEF | l MedFG | -4,48,-12 | 911 | -4.06 |
|  |  | raPFC | r cerebellum | 8,-58,-20 | 303 | -3.31 |
|  | **Slow-5** | r FEF | l MedFG | -4,50,-12 | 542 | -4.78 |
|  |  |  | l cerebellum | -36,-72,-34 | 398 | 4.43 |
|  |  |  | r thalamus | 10,-22,12 | 323 | 3.38 |
|  |  |  | r MTG | 40,-72,26 | 235 | -4.09 |
|  |  | dmPFC | l ACG | -8,36,-6 | 249 | -3.94 |
|  |  | raPFC | l precuneus | 0,-68,18 | 418 | 3.51 |
|  |  |  | r cerebellum | 6,-58,-20 | 375 | -4.34 |
|  |  | r MTG | r cerebellum | 8,-82,-34 | 276 | -3.21 |
|  |  |  | r thalamus | 18,-24,-10 | 259 | -5.48 |
|  | **Slow-4** | r FEF | r SFG | 6,66,-6 | 343 | -4.42 |
|  |  | laPFC | l PrecG | -36,20,68 | 245 | 4.20 |
| **IC Lesions** | **SFB** | l FEF | r MFG BA46 | 54,30,26 | 498 | -4.49 |
|  |  | r FEF | r CG BA31 | 10,-46,38 | 1048 | 4.37 |
|  |  |  | r IPL | 56,-50,52 | 505 | 3.81 |
|  |  | r MTG | l SPL | -38,-58,54 | 587 | -4.24 |
|  | **Slow-5** | l FEF | r MFG | 50,28,34 | 274 | -3.54 |
|  |  | laPFC | r CG BA31 | 6,-40,42 | 262 | -4.69 |
|  |  | r MTG | l SPL BA7 | -36,-58,54 | 396 | -4.23 |
|  | **Slow-4** | l FEF | r MFG BA46 | 48,19,23 | 473 | -5.33 |
|  |  | r FEF | r PC BA7 | 14,-48,40 | 1764 | 4.89 |
|  |  |  | l IFG | -22,14,-24 | 269 | -3.47 |
|  |  | r MTG | l SPL BA7 | -38,-60,52 | 523 | -3.86 |

Correlations adjusted for age, GM z-scores and FD, corrected for multiple comparisons at p<0.01. Abbreviations: ACG=anterior cingulate gyrus, BA=Broadmann area, CG=cingulate gyrus, CL=cortical lesions, dmPFC=dorso medial prefrontal cortex, FEF=frontal eye field, IC=intracortical, IFG=inferior frontal gyrus, IPL=inferior parietal lobule, l= left, laPFC=left anterior prefrontal cortex, MedFG=medial frontal gyrus, MFG=middle frontal gyrus, MTG=middle temporal gyrus, PC=precuneus, PrecG=precentral gyrus, raPFC=right anterior prefrontal cortex, r=right, SFG=superior frontal gyrus, SPL=superior parietal lobule.

**Supplementary Table 3.** Correlation between voxelwise variability and CL volumes in the standard frequency band (0.01-0.10 Hz), Slow-5 (0.01-0.027 Hz) and Slow-4 (0.027-0.073 Hz).

| **Lesions** |  | **Cluster** | **x, y, z MNI** | **Size** | **T** |
| --- | --- | --- | --- | --- | --- |
| **CLs** | **SFB** | l SFG | -6,-12,78 | 755 | 3.62 |
|  |  | l CG | -4,-40,36 | 169 | 4.06 |
|  | **Slow-5** | r CG | 14,4,26 | 675 | 3.65 |
|  | **Slow-4** | l CG | 0,0,28 | 683 | 3.93 |
|  |  | l CG | -4,-40,36 | 206 | 4.11 |

Correlations adjusted for age, GM z-scores and FD, corrected for multiple comparisons at p<0.01. Abbreviations: CG=cingulate gyrus, CL=cortical lesions, l= left, r= right, SFG=superior frontal gyrus.

**Supplementary Table 4.** Correlation between voxelwise functional connectivity and GM variability in PP patients for the standard frequency band (0.01-0.10 Hz), Slow-5 (0.01-0.027 Hz) and Slow-4 (0.027-0.073 Hz).

|  | **Seed ROI** | | **Target Cluster** | **x, y, z MNI** | **Size** | **T** |
| --- | --- | --- | --- | --- | --- | --- |
| **SFB** | | l FEF | r cerebellum | 10,-66,-26 | 1916 | -5.89 |
|  | |  | l insula | -36,10,-6 | 514 | 5.30 |
|  | |  | l MFG | -44,-2,42 | 414 | 6.00 |
|  | |  | l MTG | -48,2,-28 | 300 | 4.76 |
|  | |  | l PG | -52,-20,20 | 292 | 5.72 |
|  | |  | r STG | 34,6,-18 | 267 | 4.00 |
|  | | dmPFC | l cerebellum | -32,-78,-52 | 283 | -5.09 |
|  | |  | l claustrum | -30,4,4 | 280 | 4.19 |
|  | | l aPFC | r cerebellum | 12,-56,-14 | 2194 | -6.45 |
|  | |  | l PrecG | -44,-16,-32 | 426 | 4.62 |
|  | |  | l cerebellum | -6,-52,-28 | 296 | -4.48 |
|  | | r aPFC | r insula | 32,-32,16 | 1936 | -7.49 |
|  | |  | r CG | 18,-36,46 | 453 | -5.35 |
|  | |  | l PHG | -22,-14,32 | 415 | 5.64 |
|  | |  | l AG | -32,-60,32 | 314 | 5.50 |
|  | |  | r PG | 40,-28,38 | 298 | -4.46 |
|  | | r MFG | r IFG | 58,22,12 | 385 | 4.73 |
|  | |  | r cerebellum | 30,-68,-44 | 353 | 4.73 |
|  | |  | r SFG | 30,48,26 | 326 | 5.64 |
| **Slow-5** | | l FEF | r cerebellum | 38,-66,-32 | 1672 | -7.46 |
|  | |  | l MTG | -48,2,-28 | 789 | 6.13 |
|  | |  | r STG | 36,-52,12 | 713 | -6.15 |
|  | |  | r PG | 26,-34,58 | 525 | -5.17 |
|  | |  | l STG | -54,-36,14 | 350 | 4.50 |
|  | |  | r PG | 38,-18,32 | 313 | -5.64 |
|  | |  | l PG | -56,-20,16 | 265 | 6.49 |
|  | |  | l uncus | -22,-4,-38 | 236 | 4.41 |
|  | | dmPFC | r SFG | 14,52,24 | 348 | 5.68 |
|  | |  | r IFG | 36,32,-2 | 246 | 5.19 |
|  | | l aPFC | r cerebellum | 14,-56,-16 | 250 | -5.18 |
|  | | r aPFC | l uncus | -24,-12,-36 | 530 | 5.97 |
|  | |  | r FG | 38,-48,-10 | 480 | -5.54 |
|  | |  | l MTG | -30,-62,30 | 265 | 5.35 |
|  | |  | l SFG | -14,-20,52 | 257 | 4.92 |
|  | |  | r STG | 42,20,-36 | 248 | 5.40 |
|  | |  | l IFG | -24,34,-24 | 239 | 5.30 |
|  | | r MFG | r cerebellum | 24,-66,-32 | 601 | 5.87 |
|  | |  | r SFG | 28,42,18 | 313 | 5.73 |
|  | | r MTG | r insula | 26,24,20 | 269 | 5.18 |
| **Slow-4** | | l FEF | r cerebellum | 20,-42,-30 | 508 | -5.04 |
|  | | dmPFC | l PG | -58,-12,20 | 416 | 5.57 |
|  | | l aPFC | r cerebellum | 18,-44,-24 | 804 | -7.07 |
|  | |  | l PG | -60,-12,20 | 423 | 5.46 |
|  | | r aPFC | r cerebellum | 18,-42,-20 | 698 | -6.37 |
|  | |  | r insula | 38,-34,18 | 544 | -6.90 |
|  | | r MFG | r cerebellum | 22,-62,-44 | 731 | 5.69 |
|  | | r MTG | l insula | -30,14,-6 | 363 | -4.76 |
|  | |  | r cerebellum | 2,-56,-10 | 285 | 5.59 |

Correlations adjusted for age, GM z-scores and FD, corrected for multiple comparisons at p<0.01.

Abbreviations: ACG=anterior cingulate gyrus, AG=angular gyrus, CG=cingulate gyrus, FEF=frontal eye field, dmPFC=dorso medial prefrontal cortex, FG=fusiform gyrus, IFG=inferior frontal gyrus, IPL=inferior parietal lobule, l= left, laPFC=left anterior prefrontal cortex, MedFG=medial frontal gyrus, MFG=middle frontal gyrus, MOG=middle occipital gyrus, MTG=middle temporal gyrus, PCG=posterior cingulate gyrus, PHG=parahippocampal gyrus, PG=postcentral gyrus, PrecG=precentral gyrus, r= right, raPFC=right anterior prefrontal cortex, SCG=subcallosal gyrus, SFG=superior frontal gyrus, STG=superior temporal gyrus.
